# Extraction of Hydroxyapatite from Camel Bone for Bone Tissue Engineering Application

**DOI:** 10.3390/molecules27227946

**Published:** 2022-11-16

**Authors:** Zohaib Khurshid, Mohammed Farhan Alfarhan, Javed Mazher, Yasmin Bayan, Paul R. Cooper, George J. Dias, Necdet Adanir, Jithendra Ratnayake

**Affiliations:** 1 Department of Prosthodontics and Dental Implantology, College of Dentistry, King Faisal University, Al-Ahsa 31982, Saudi Arabia; 2 Department of Oral Science, Faculty of Dentistry, University of Otago, 310 Great King Street, Dunedin 9016, New Zealand; 3 Department of Surgery, College of Medicine, King Faisal University, Al-Ahsa 31982, Saudi Arabia; 4 Department of Physics, College of Science, King Faisal University, Al-Ahsa 31982, Saudi Arabia; 5 Department of Anatomy, University of Otago, 310 Great King Street, Dunedin 9016, New Zealand; 6 Department of Restorative Dentistry, College of Dentistry, King Faisal University, Al-Ahsa 31982, Saudi Arabia

**Keywords:** camel, biowaste, environmental sustainability, bone, hydroxyapatite, graft, tissue engineering

## Abstract

Waste tissues such as mammalian bone are a valuable source from which to extract hydroxyapatite. Camel bone-based hydroxyapatite (CBHA) was extracted from the femur of camel bones using a defatting and deproteinization procedure. The extracted CBHA was mechanically, chemically, physically, morphologically and structurally characterized. Fourier-Transform Infra-Red (FTIR) spectra, Micro-Raman, and X-ray diffraction analysis confirmed successful extraction of hydroxyapatite. The mechanical properties of the CBHA scaffold were measured using a Universal Instron compression tester. Scanning electron microscopy showed the presence of a characteristic interconnected porous architecture with pore diameter ranging from 50–600 µm and micro-computer tomography (Micro-CT) analysis identified a mean porosity of 73.93. Thermogravimetric analysis showed that the CBHA was stable up to 1000 °C and lost only 1.435% of its weight. Inductively coupled plasma–mass spectrometry (ICP-MS) and Energy-dispersive-X-ray (EDX) analysis demonstrated the presence of significant amounts of calcium and phosphorus and trace ions of sodium, magnesium, zinc, lead and strontium. Following 21 days of incubation in simulated body fluid (SBF), the pH fluctuated between 10–10.45 and a gradual increase in weight loss was observed. In conclusion, the extracted CBHA is a promising material for future use in bone tissue regeneration applications.

## 1. Introduction

In order to consider a material for use as a bone graft its properties need to be well understood. This includes the characterization of its chemical composition, mechanical and biological properties. Bone graft materials are developed with the aim of replacing missing or lost bone tissue. An optimal bone graft material will promote host bone-tissue remodeling and regeneration, leading to full replacement of bone over time [1]. Autografts have long been considered the gold standard for bone graft materials due to their superior characteristics. They are osteogenic, osteoconductive, osteo-inductive and osteo-integrative [2] and they exhibit minimal risk of disease transmission or induction of an adverse immune response as the donor tissue is host derived [2]. However, there remain significant drawbacks in the use of autografts, such as their limited availability, especially when intended for larger bone-defect treatment. Donor site morbidity is a further limitation and an added risk factor for the patient [3,4]. Xenografts are bone substitute materials derived from non-human species such as the horse, pig and cow [5]. The use of xenografts removes patient risk of donor site morbidity as only a single surgery is required to place the bone graft. Furthermore, treatments can be made more widely available and tailored for a range of applications, shapes and sizes enabling versatility for complex and large bone defect treatments [6].

Bone is a biological hard tissue with a complex composition. The inorganic component comprises the majority (~70%) of the tissue and hydroxyapatite is the primary constituent [7]. Hydroxyapatite has important properties which make it desirable as a bone graft material, including its biocompatibility, osteo-conductivity and bioactivity, it is also not associated with adverse immunological reactions or induction of inflammatory processes [8]. Hydroxyapatite (HAp) is the main mineral found in bones and teeth with a chemical formula of Ca_10_(PO_4_)_6_(OH)_2_. It can be obtained synthetically or derived from natural sources. Extensive research reports have demonstrated that biologically derived hydroxyapatite is a valuable candidate to produce effective and inexpensive xenografts for bone regeneration [9]. Production of synthetic hydroxyapatite requires strict control of parameters such as composition and purity of the starting material, Ca/P mole ratio, pH value and the temperature of the solutions. On the whole, producing synthetic hydroxyapatite is a laborious and costly process. A lower yield is extracted from the starting material when hydroxyapatite is produced synthetically. According to Bahrololooma et al. approximately 1 kg of hydroxyapatite is produced when 1.6 kg of cortical bone is used [10]. Hence, extracting hydroxyapatite from natural sources such as camel bone adds further economic value and it is environmentally preferable over synthetic hydroxyapatite [11]. The mineral component of human bone has a non-stoichiometric carbonated hydroxyapatite with trace amounts of ions such as carbonate (CO_3_^2−^), sodium (Na^+^), magnesium (Mg^2+^), iron (Fe^2+^), fluoride (F^−^), silicate and chloride (Cl^−^) [1]. These trace elements play a vital role in bone metabolism and osteointegration. Moreover, these vital trace elements are absent when hydroxyapatite is produced synthetically. Consequently, the use of naturally derived hydroxyapatite sources is attractive, and dense cortical bone, cancellous or trabecular bone, which have a relatively large porosity, make it suitable for osseointegration [12].

In the Kingdom of Saudi Arabia, camel bone is a waste material resource which can be commercially obtained from the food industry and local abattoirs [13,14,15]. Due to cultural and religious reasons, bone graft materials derived from some species, such as pigs and cows, may not be acceptable for clinical application. Consequently, camels may serve as an alternative mammalian bone source to generate bone graft materials. As camel bones as biowaste are readily accessible and relatively inexpensively obtained, we hypothesized that they would provide an excellent source for the extraction of hydroxyapatite (HAp) for use as a xenograft material [5]. Consequently, the aim of this study was to extract HAp from camel (*Camelus dromedarius*) bone using defatting and deproteinization procedures, and to characterize its physiochemical properties using a variety of analytical techniques.

## 2. Materials and Methods

### 2.1. Camel Bone Preparation

**Figure 1:**
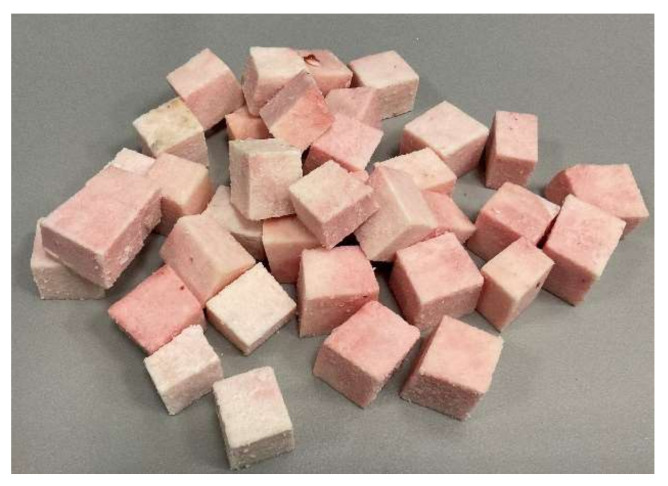
Fresh and clean camel bone dissected into cubes (2 cm × 2 cm × 2 cm).

The camel (*Camelus dromedarius*) bones were collected from a local slaughterhouse in Al-Ahsa, Saudi Arabia. The bones were washed using distilled water, and carefully cleaned to remove all fats, meat and blood debris using a high-powered water jet. After cleaning, the bones were cut into blocks using a bone saw and further trimmed into cubes using the MT plus Dental Plaster (Wet & Dry) Trimmer (Figure 1) (Renfert, Hilzingen, Germany).

### 2.2. Defatting and Deproteinization

The bone cubes were heated for 2 h at 15 psi (103.4 kPa) in a domestic pressure cooker to remove the bone marrow fats and lipids. The water ratio was set as previously described by Ratnayake et al., (2017) at 50 mL of water per one bone cube [16]. After boiling in the pressure cooker, the bone cubes were washed with deionized water and cleaned with a POWER steamer 2 High Pressure (Wet & Dry) Steam Cleaner (Renfert, Hilzingen, Germany). Prepared bone cubes were dried in a desiccator for 2 h and then soaked in a 0.1 M NaOH (Sigma Aldrich, St. Louis, MO, USA) solution for 16 h (NaOH solution was changed after 8 h) at 70°C. To ensure removal of the bone marrow fats and lipids, the bone cubes were microwaved in a domestic oven (220 V 60 Hz, 1100 W, ClassPro^®^, Beijing, China) for 5 min. Finally, the blocks were dried in an oven at 50 °C for 30 min prior to deproteination.

Low heat sintering in a muffle furnace (Thermolyne, Burlington, IA, USA) was used for deproteinization of camel bone cubes for 8 h at 650 °C. Subsequently, blocks were ground manually using a mortar and pestle prior to chemical analysis. Representative intact blocks were preserved for use in morphological analysis.

### 2.3. Characterization

#### 2.3.1. Fourier-Transform Infra-Red Spectroscopy (FTIR)

The functional groups present in the CBHA was determined using Fourier-transform infra-red spectroscopy (FTIR). The FTIR spectra was conducted using a Perkin Elmer Fourier-transform spectrophotometer (Merck KGaA, Darmstadt, Germany) in the mid-infra-red region between the frequency range of 400–4000 cm^−1^. A background scan was obtained each time and both the background and sample scan time was 24 scans with measurement time >30 s. The spectra were obtained at a resolution of 4 cm^−1^.

#### 2.3.2. Micro-Raman Analysis

A finely powdered sintered CBHA sample was used in the micro-Raman spectroscopy to study the effects of the alkaline treatment on the structural and compositional behavior of the hydroxyapatite phase. All the Raman spectra were acquired using He-Cd gas laser at fixed laser power of 300 mW in the confocal geometry of the Raman spectrometer (Horiba Labram Evolution-2; Longjumeau, France). The excitation wavelength of the laser was fixed at 448 nm and the Raman signals were collected from the 100× microscopic objective lens through 100 µm diameter confocal hole. The Labspec-6 software (Horiba, Longjumeau, France) was used in the Raman data acquisition and spectrum plotting.

#### 2.3.3. XRD Analysis

X-ray diffraction analysis was conducted to determine the phase composition and crystal structure of the CBHA scaffold using an X-ray diffractometer (PANalytical X1Pert PRO MPD System (PW3040/60)) in the region 10° < 2θ < 70° using Cu-Kα radiation. The generator was set to 40 kV and 40 mA.

#### 2.3.4. Scanning Electron Microscopy (SEM)

Pore size measurements as well as surface morphology analysis were conducted using a scanning electron microscope (Oxford JOEL 2300; Tokyo, Japan). The instrument was equipped with energy dispersive X-ray (EDX) functioning at 15 kV and 15 mA (EDX; Oxford, UK). The samples were dissected using a scalpel blade to expose the internal morphology for analysis then sputter-coated with gold and palladium for the EDX analysis.

#### 2.3.5. Inductively Coupled Plasma Mass Spectrometry (ICPMS)

An Agilent 7500 cs quadrupole analyzer was used to conduct inductively coupled plasma–mass spectroscopy (ICP-MS) on the sample. This was performed to determine the chemical composition and to obtain the Ca/P ratio of the hydroxyapatite extracted. These findings were then compared with the results from the EDX analysis.

#### 2.3.6. Micro CT Analysis

An 8 mm CBHA scaffold was prepared using a biopsy punch. Analysis of the microscopic architecture of the CBHA scaffold was undertaken using a Bruker SkyScan 1172 Hi-Res micro-CT scanner (Bruker-Micro CT, Kontuch, Belgium). The size of the focal spot was chosen at <5 μm, the voltage was 30 kV, and the current 175 μA. To obtain 2D images of the 3D sample, it was rotated by 180° at 0.4° per sector. The pixel size of the camera was 11.4 μm and >1000 images were captured. SkyScan software based on the “Advanced Porosity Analysis” (Bruker microCT; Berlin, Germany) was used to analyze all the 2D images taken. The μCT-analyzer software was used for 3D analysis to determine the porosity of the structure. Porosity was reported as a percentage for pore size of the scaffold.

#### 2.3.7. Thermogravimetric Analysis (TGA)

Thermal degradation of the sample was investigated using a TGA analyzer (Q50, TA instruments) within an N_2_ atmosphere from 20 °C to 1000 °C at a heating rate of 10 °C per minute.

#### 2.3.8. Chemical Stability and Biodegradation in Simulated Body Fluid (SBF)

To study the degradation of the hydroxyapatite sample, a stock solution of simulated body fluid (SBF) was prepared according to the Kokubo protocol [17]. The sample was cut using a scalpel blade into similar volume cubes and weighed (*n* = 3). The samples were incubated in 10 mL of SBF in Falcon tubes at 37 ± 1 °C for 1, 3, 7, and 21 days. After each time period, the pH was measured using a pH conductometer (Ionode, Acorn Scientific Ltd., Auckland, New Zealand). The hydroxyapatite samples were then carefully extracted from the solution and completely dried in an incubator at 37 °C for 24 h before measuring the dry mass again. The weight change was calculated as a percentage following the formula: W_L_ = (W_0_ − W_1_)/W_0_ × 100%.

#### 2.3.9. Mechanical Properties

The mechanical properties of the CBHA scaffold were measured using a Universal Instron compression tester. Bovine-derived hydroxyapatite (BHA) scaffolds were used as a control. The scaffolds were analyzed on cubic samples with dimensions of ~10 mm × ~10 mm × ~10 mm. Before testing, to ensure the opposite faces remained parallel to each other, samples were fine-sanded using 800-grit sandpaper to flatten the trabecular struts of the scaffolds. For compression of the samples, a 1 kN load cell was operated at a crosshead speed of 0.5 mm/min in the downward direction. The extension was measured via the Instron’s internal extensometer until catastrophic brittle failure of the samples occurred. The Young’s modulus was determined using the compression stress–strain curve with the Exponent software (version v6.1.5.0, Menlo Park, CA, USA). The Young’s modulus was calculated from the most linear portion of the stress–strain curve. Fifteen replicates were tested, and the yield strength was calculated from the load–deformation curve at the yield point. A two-tail student’s *t*-test was performed to determine the statistical significance (*p* < 0.05) of the differences in mechanical properties of each material [18].

## 3. Results

### 3.1. Bone Processing

**Figure 2:**
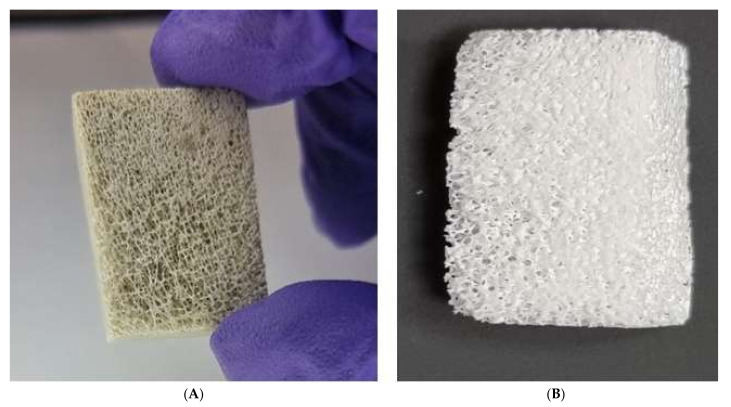
(**A**) Bone cubes after defatting and (**B**) Bone cubes after sintering.

After defatting, the bone cubes changed from a light brown to white color, and the pores were more apparent (Figure 2A). This supported the success of the cleaning steps applied. Following sintering, the bone cubes became whiter and harder (Figure 2B).

### 3.2. Fourier-Transform Infrared Spectroscopy (FTIR)

**Figure 3:**
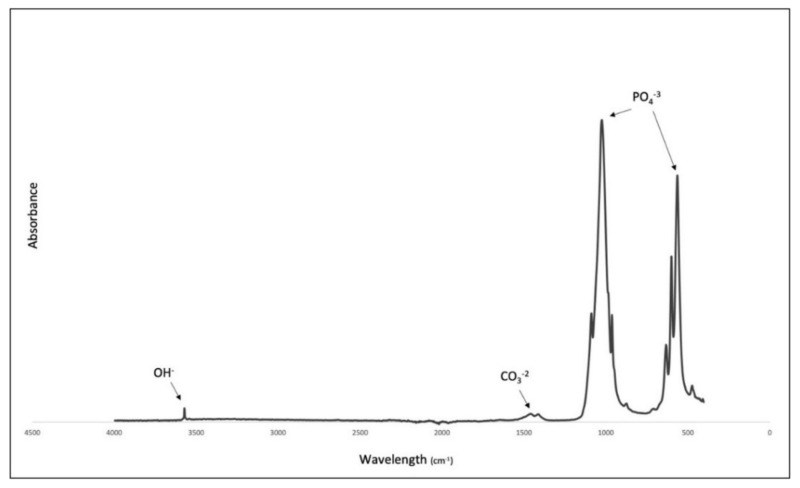
FTIR spectra of CBHA.

The spectra obtained from FTIR analysis (Figure 3) showed distinct bands associated with phosphate, hydroxyl and carbonate groups. These data indicate the successful removal of fat, collagen and other organic matter leaving only the hydroxyapatite with retained carbonate [8,16].

### 3.3. X-ray Diffraction (XRD)

**Figure 4:**
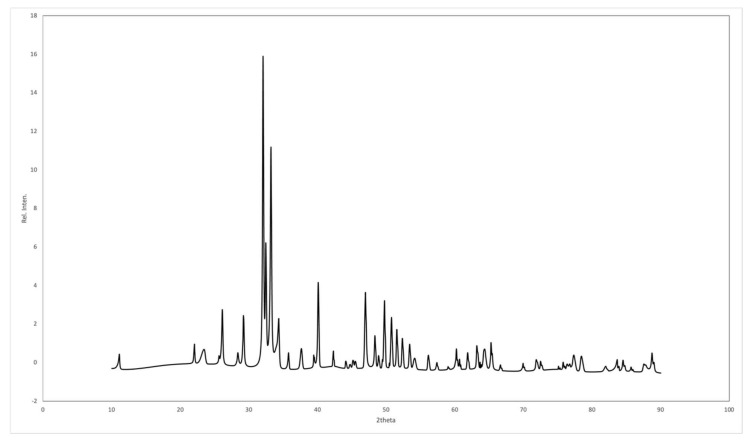
XRD patterns of the sintered CBHA powder.

The XRD pattern for the sintered CBHA is shown in Figure 4. XRD profiles for the calcined CBHA were similar and corresponded with the phase pure-HA characterized pattern (JCPDS pattern 09-0432).

A defined peak was observed at 3570 cm^−1^ associated with the presence of hydroxyl (OH^−^). Phosphate bands were appreciated at 1092 to 1040 cm^−1^, 962 cm^−1^, 633–566 cm^−1^ and 473 cm^−1^, respectively. Carbonate was retained within the CBHA sample as confirmed by the bands present in the region of 1455 to 1418 cm^−1^ and at 873 cm^−1^.

### 3.4. Chemical Compositional Analysis by Micro-Raman Spectroscopy

**Figure 5:**
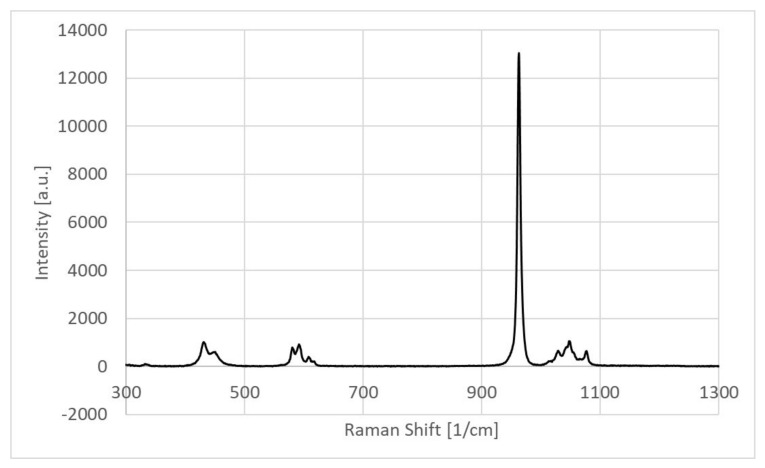
Micro-Raman spectra of the CBHA powder.

An intense Raman peak corresponded with the stretching vibration mode of the phosphate group (PO_4_^3−^) and was observed in the spectrum. As shown in Figure 5, at 963 cm−1 the peak indicated the formation of the hydroxyapatite compositional phase in the sample [8,19,20]. Moreover, three additional phosphate-related Raman peaks were observed at 434 cm^−1^, 590 cm^−1^ and 1032 cm^−1^ corroborating that the main phase of the sample was hydroxyapatite composite [8,19]. Other Raman peaks at 1046 cm^−1^ and 1074 cm^−1^ represented the presence of C-H and C-O modes of vibrations, respectively, in the CBHA sample [19,21].

### 3.5. Scanning Electron Microscopy

**Figure 6:**
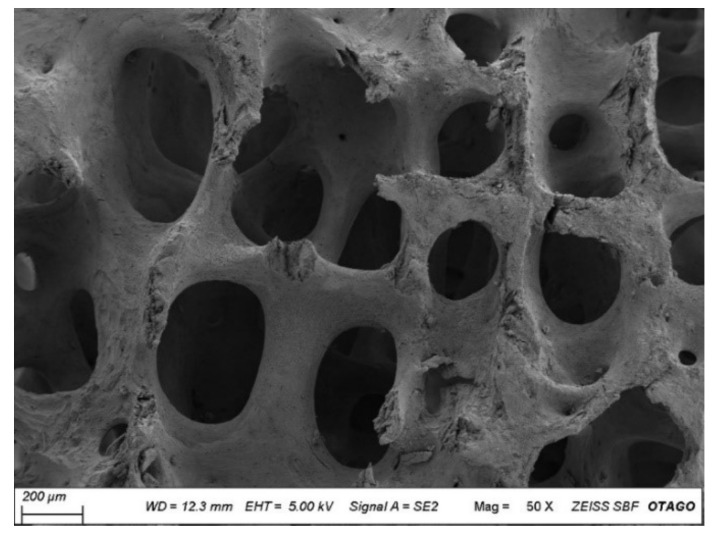
Representative scanning electron micrograph (SEM) of CBHA. Scale Bar = 200 µm.

The scanning electron micrograph revealed the porous structure of the CBHA scaffold. The pore size ranged from 50–600 μm. As shown in Figure 6, there was some fracture evident within the structure as well as residual debris. The uniform porous arrangement indicated that the organic material was successfully removed during the defatting and deproteinization procedures.

### 3.6. Micro-Architectural Analysis of the CBHA Scaffold

**Figure 7:**
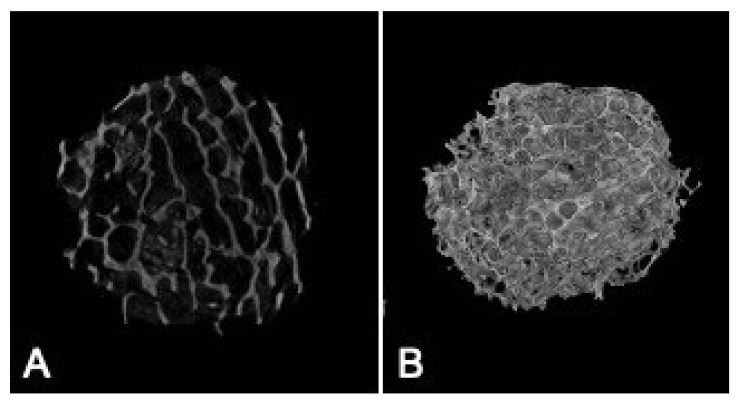
Representative μ-CT images of the CBHA scaffold. (**A**) The 2D cross section of the BHA scaffold; (**B**) The 3D rendering of the CBHA scaffold.

Approximately 800 2D images were generated from the μ-CT analysis of the central area of the 8 mm CBHA scaffold (Figure 7A). Figure 7B illustrates the 3D architecture of the CBHA scaffold exhibiting its interconnected porous structure. The SKYSCAN software calculated the total porosity of the CBHA scaffold to range from 72.35% to 75.55%.

### 3.7. Energy Dispersive X-ray (EDX) and Inductive Coupled Mass Analysis

**Figure 8:**
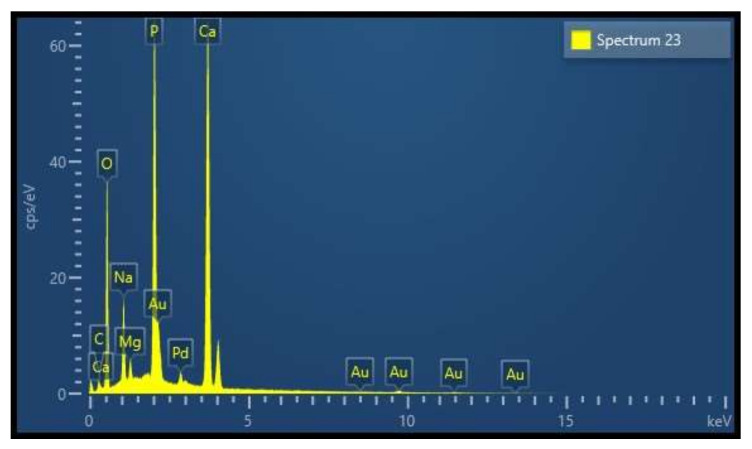
EDX analytical spectra of the CBHA sample.

EDX analysis revealed the elemental composition of the CBHA sample to be primarily composed of calcium and phosphorous (Figure 8) with trace amounts of sodium and magnesium. The Ca: P ratio in Table 1 was in agreement with data from the EDX and ICP-MS analysis. Table 1 shows that the concentration of toxic elements, including Cd, As and Pb, were all below the American Society for Testing and Materials (ASTM) maximum limit suggested (F1185-03).

### 3.8. Thermal Gravimetric Analysis

**Figure 9:**
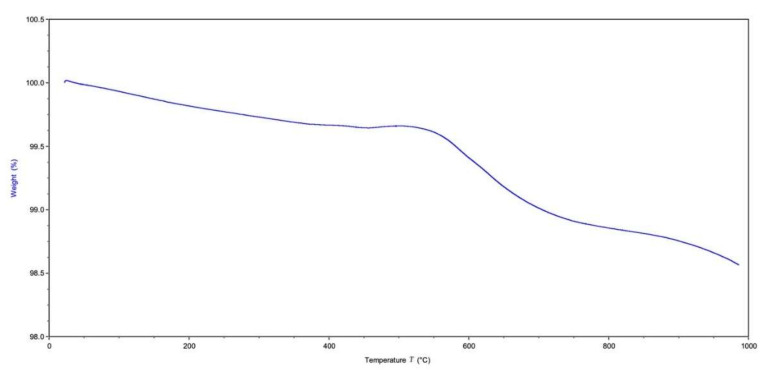
Graphical results of thermogravimetric analysis (TGA).

The results of the thermal gravimetric analysis is represented in Figure 9. There was no significant weight loss detected. The initial weight loss detected from 50 °C to 200 °C was associated with the evaporation of residual water absorbed in the sample. As seen in the results from the FTIR analysis, there was a small amount of carbonate remaining with the sample. The small weight loss (~0.2 wt.%) associated with the temperature increase from 250 °C to 400 °C was associated with the thermal decomposition of this remaining carbonate. The majority of the weight loss was seen from the temperature range change from 500 °C to 1000 °C indicating the materials’ thermal stability. The total weight loss percentage of the sample was ~1.435 wt.%.

### 3.9. Chemical Stability and Degradation

**Figure 10:**
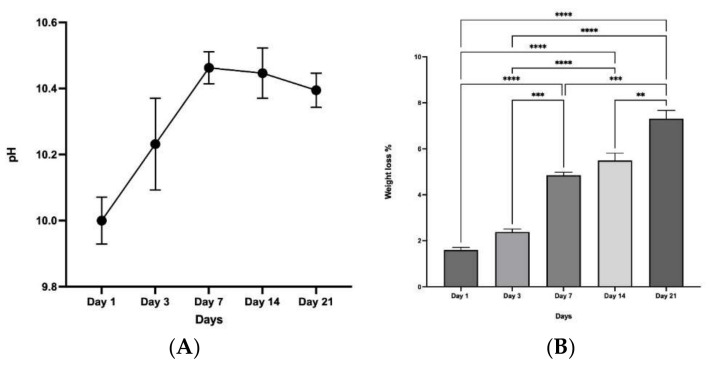
(**A**) Graphical representation of pH change over time of incubation in SBF (*n* = 3). (**B**) The weight loss of the CBHA scaffolds as a function of time in SBF. (*n* = 3. ** *p* < 0.01, *** *p* < 0.001, **** *p* < 0.0001, error bars represent +SE of the mean).

Prior to immersion and soaking of the CBHA scaffolds, the pH of the SBF solution (day 0) was recorded at 7.4. As a control, a SBF solution without the sample was used which exhibited a stable pH throughout the experimental period. In experimental samples, after the first day, the pH rose to a value of 10 and increased to 10.45 by day 7. After 7 days, the pH value gradually decreased to 10.3 by day 21 (Figure 10A). The CBHA scaffold macroscopically retained its original shape after soaking in SBF for 21 days, which indicated that the scaffold had excellent structural stability. According to the data (Figure 10B), the weight loss percentage increased up to day 21, significantly (ANOVA, *p* < 0.0001).

### 3.10. Mechanical Testing

**Figure 11:**
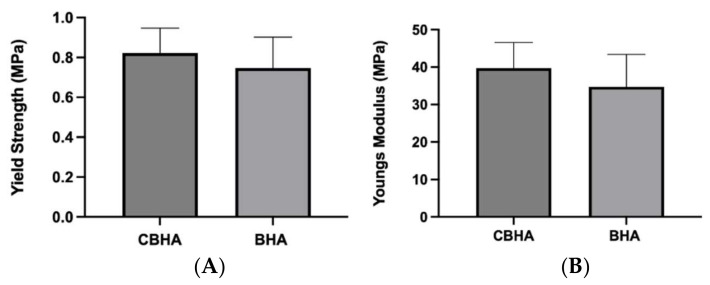
Mechanical properties of the CBHA and BHA scaffold. (**A**) Yield strength (*n* = 15, *p* = 0.7068). (**B**) Young’s modulus (*n* = 15, *p* = 0.6547). Bar graphs were reported as ± standard error of the mean (S.E.M).

The mechanical properties of the CBHA and BHA scaffolds are shown in Figure 11. Although the yield strength and Young’s modulus were numerically higher in the CBHA scaffold, there was no statistically significant difference in the mechanical properties (yield strength and Young’s modulus) compared to the BHA scaffold.

## 4. Discussion

This study, for the first time, demonstrated the extraction of hydroxyapatite from cancellous bone cubes derived from camels’ femurs following a successful defatting and deproteination procedure adapted from Ratnayake et al., (2017) [16]. Initially, to remove the potential for immunogenicity, bone cubes were pressure-cooked to remove lipids and organic materials in order to create CBHA. Following a NaOH solution treatment, microwave heating was utilized to ensure that all organic and lipid residues were eliminated from the bone cubes’ interior pores [22]. Previous studies have shown that sintering the bone cubes at a low temperature effectively deproteinized bone samples without affecting their characteristics, such as crystallinity and phase purity [16,23]. Therefore, the camel-bone cubes underwent this low-temperature heat treatment, changing from brown to grey for the first two hours before turning chalky white (Figure 1 and Figure 2) which confirms the elimination of the organic matter. Results from the FTIR analysis confirmed the presence of hydroxyapatite [18]. The FTIR findings were similar to those generated for hydroxyapatite sourced from other natural sources, such as bovine bone and teeth [8,16,18]. Furthermore, there were no peaks associated with residual fat and collagen in the specimens suggesting all organic materials including collagen and fat were successfully removed during bone processing [16].

Raman spectrum results also strongly supported the presence of the hydroxyapatite phase since all the major Raman vibrational modes of the sample were compared with the high-quality spectral data obtained from the RRUFF hydroxyapatite database (ID number R100225). Indeed, a direct sample peak match was obtained with that of the Ca_5_(PO_4_)_3_ (OH)_2_ reference peaks [24]. Furthermore, Timlin et. al. observed that typically the stoichiometric hydroxyapatite samples showed a stretching vibration mode at 1031 cm^−1^ since it is Raman-active whilst the non-stoichiometric phase is usually Raman-inactive [19]. Thus, the presence of the 1032 cm^−1^ Raman peak in our sample indicates the stoichiometric retention of the hydroxyapatite phase. The obtained XRD spectra for BDHA was compared with the JCPDS pattern 09-0432 and all the peaks corresponded with hydroxyapatite (Figure 4). The XRD diffraction patterns for the BDHA showed sharp and narrow peaks, indicating an increase in crystallinity. Secondary phases such as tricalcium phosphate (β-TCP) (2theta = 31.13°) and calcium oxide (CaO) (2theta = 37°) were not detected which further confirms the proposed application of this method to produce pure hydroxyapatite from camel bone.

Scanning electron microscopy (SEM) analysis allows for the determination of pore size and surface architecture of the hydroxyapatite scaffold material. A minimum pore size of 50–500 μm is needed for successful osseointegration and osteogenesis through activities such as cell migration, cell differentiation and cell proliferation [25,26]. Osteogenic cells need to migrate and attach to the surface of the scaffold to allow for bone growth throughout the porous structure. Furthermore, the pores act as spaces for angiogenesis which enables nutrient provision thus supporting bone growth [27]. The results of the SEM show a pore size ranging from 50 to 600 μm which is therefore supportive of potential osteogenesis and osseointegration properties. Notably, the pore size range reported by producers of Bio-Oss^®^, a bovine-derived porous bone material is 250–1000 μm [28,29]. Therefore, the pore size of the CBHA is within a similar range of that of a widely used commercial product. Porosity also plays an important role in bone formation, as it facilitates cell motility, proliferation and differentiation [27]. The porosity of the CBHA scaffold was also similar to the porosity of human cancellous bone (>70%) which suggests that the CBHA scaffold is suitable for bone tissue regeneration allowing the exchange of nutrients and metabolic waste. Several studies have confirmed that a porous material would enhance osteogenesis compared to a non-porous material [30,31,32]. Furthermore, in applications such as oral surgery, a porous material improves the mechanical interlocking between the implant and bone, providing excellent mechanical stability [30].

Findings from both ICP-MS and EDX indicate that the CBHA material is primarily composed of calcium and phosphorous as well as trace elements including sodium, magnesium, zinc and strontium. These elements are known to be important for promoting bone metabolism. Magnesium plays a role in stimulating osteoblast proliferation and differentiation during the early stages of osteogenesis [33]. Magnesium deficiencies can adversely affect bone growth and osteoblastic activity. Sodium is important for bone mineralization, cell adhesion and inhibiting bone resorption [33]. Similarly, strontium also reduces bone resorption and osteoclastic activity [33]. In vitro and in vivo studies conducted on synthetic HA doped with Sr ions has shown desirable results in increasing the bioactivity of HA [34]. On the other hand, carbonate is the most abundant impurity element found in apatites and plays a vital role in bone metabolism. In addition, carbonated HA significantly reduces the thermal stability resulting in decomposition at relatively low temperatures in the region of 700 °C. However, in this study, a sintering temperature of 650 °C was used and secondary phases such as CaO were not observed (Figure 4). The ratio of calcium to phosphorous was calculated at 1.79 from SEM-EDX and 1.76 from ICP-MS; however, the stoichiometric Ca: P ratio of hydroxyapatite ranges from 1.5 to 1.65. The findings from the CBHA analysis are likely higher due to the presence of retained carbonate and trace amounts of sodium and magnesium in the samples.

TGA showed that the sample was thermally stable as there was a loss of only ~1.4% of the original weight. The initial loss (50–200 °C) was from the evaporation of residual water. The minimal weight loss observed between 250–600 °C (exothermic loss) was a result of the decomposition of the carbonate groups in the CBHA scaffold as observed by the FTIR analysis (Figure 3). Notably, there was insignificant weight loss above 600 °C suggesting that the CBHA is thermally stable. Simulated body fluid (SBF) was used to mimic the extracellular effect of human body fluids [17]. The solubility of CBHA scaffolds was investigated by measuring the changes in pH over a 21-day incubation time. The increase in the pH (up to day 14) was mainly attributed to the OH^−^ and Ca^2+^ ions leaching out from the HA lattice into the solution. However, potentially this could also be due to the carbonate groups in the CBHA decomposing into CaO to react with water to form Ca(OH)_2_. Once the Ca^2+^ and OH^−^ are limited, the pH subsequently decreases. In this study, the CBHA scaffold was structurally stable, as the scaffold retained its original shape. However, according to Figure 10B, the weight loss percentage increased during the 21-day period which suggests the degradation of the scaffold. This phenomenon was likely mainly a result of the interconnected porous architecture and the higher porosity which the CBHA scaffold possesses.

The CBHA scaffold is derived from the femoral condyle portion of the camel and consists of cancellous bone. Past studies have reported that synthetic hydroxyapatite exhibits superior mechanical properties exhibiting a Young’s modulus and yield strength value in the range of 35–120 GPa and 3–5 MPa, respectively [35,36]. However, the CBHA possessed relatively poor mechanical properties and thus is intended to be use as a bone substitute for minor bone augmentation and extraction socket management in regenerative dentistry. The CBHA scaffold is derived from the femoral condyle portion of the camel and consists of cancellous bone. The organic matrix is completely removed through a variety of defatting and deproteinization steps, leaving behind the mineral portion. Although the resulting bone is strong, it has a brittle nature which limits its use as a xenograft in areas subjected to mechanical loading. The CBHA consists of an interconnected porous architecture (Figure 6 and Figure 7) which reduced the strength. The blending of CBHA with polymeric and antibacterial material could be great for more successful bone tissue engineering graft is possible and promising [37,38].

## 5. Conclusions

This study revealed the potential for using biowaste for translational research for the treatment of bone defects in orthopedics such as total joint replacement and dental surgeries. Notably, a xenograft material was developed using waste camel bone using a relatively simple, reproducible and cost-effective approach. The physicochemical properties of the CBHA scaffold were successfully characterized using a number of established analytical techniques. However, further in vitro and in vivo studies are necessary to evaluate the biocompatibility of the CBHA scaffold for clinical applications.

## Figures and Tables

**Table 1:** Chemical composition of the hydroxyapatite sample obtained via ICP-MS analysis.

Sample	Na	Mg	P	Ca	Zn	Sr	Cd	As	Pb
**Hydroxyapatite (mg/kg)**	2.90 × 10^4^	6.73 × 10^3^	1.58 × 10^5^	3.6 × 10^5^	198	880	<0.25	<0.25	3.20
**ASTM Maximum Limits (F1185-03)**							5	211	30
Ca: P (SEM-EDX) Ca: P (ICP-MS)	1.79 1.76								

## Data Availability

Not applicable.

## References

[1] Zhao R., Yang R., Cooper P.R., Khurshid Z., Shavandi A., Ratnayake J. (2021). Bone grafts and substitutes in dentistry: A review of current trends and developments. Molecules.

[2] Chiarello E., Cadossi M., Tedesco G., Capra P., Calamelli C., Shehu A., Giannini S. (2013). Autograft, allograft and bone substitutes in reconstructive orthopedic surgery. Aging Clin. Exp. Res..

[3] Misch C.M. (2010). Autogenous bone: Is it still the gold standard?. Implant Dent..

[4] Ratnayake J.T.B., Mucalo M., Dias G.J. (2017). Substituted hydroxyapatites for bone regeneration: A review of current trends. J. Biomed. Mater. Res. Part B Appl. Biomater..

[5] Mohd Pu’ad N.A.S., Koshy P., Abdullah H.Z., Idris M.I., Lee T.C. (2019). Syntheses of hydroxyapatite from natural sources. Heliyon.

[6] Damien C.J., Parsons J.R. (1991). Bone graft and bone graft substitutes: A review of current technology and applications. J. Appl. Biomater..

[7] Sommerfeldt D., Rubin C. (2001). Biology of bone and how it orchestrates the form and function of the skeleton. Eur. Spine J..

[8] Jaber H.L., Hammood A.S., Parvin N. (2018). Synthesis and characterization of hydroxyapatite powder from natural Camelus bone. J. Aust. Ceram. Soc..

[9] Moore W.R., Graves S.E., Bain G.I. (2001). Synthetic bone graft substitutes. ANZ J. Surg..

[10] Bahrololoom M., Javidi M., Javadpour S. (2009). Characterisation of natural hydroxyapatite extracted from bovine cortical bone ash. J. Ceram. Process. Res..

[11] Barakat N.A.M., Khil M.S., Omran A.M., Sheikh F.A., Kim H.Y. (2009). Extraction of pure natural hydroxyapatite from the bovine bones bio waste by three different methods. J. Mater. Process. Technol..

[12] Sikavitsas V.I., Temenoff J.S., Mikos A.G. (2001). Biomaterials and bone mechanotransduction. Biomaterials.

[13] (2018). GAS General Information about The Kingdom of Saudi Arabia.

[14] Al-Kanhal M.A., Al-Mohizea I.S., Al-Othaimeen A.I., Khan M.A. (1999). Nutritive value of various rice based dishes in Saudi Arabia. Ecol. Food Nutr..

[15] Kurtu M.Y. (2004). An Assessment of the Productivity for Meat and the Carcass Yield of Camels (*Camelus dromedarius*) and of the Consumption of Camel Meat in the Eastern Region of Ethiopia. Trop. Anim. Health Prod..

[16] Ratnayake J.T.B., Gould M.L., Shavandi A., Mucalo M., Dias G.J. (2017). Development and characterization of a xenograft material from New Zealand sourced bovine cancellous bone. J. Biomed. Mater. Res. Part B Appl. Biomater..

[17] Kokubo T., Takadama H. (2008). Simulated Body Fluid (SBF) as a Standard Tool to Test the Bioactivity of Implants. Handbook of Biomineralization: Biological Aspects and Structure Formation.

[18] Ratnayake J.T., Ross E.D., Dias G.J., Shanafelt K.M., Taylor S.S., Gould M.L., Guan G., Cathro P.R. (2020). Preparation, characterisation and in-vitro biocompatibility study of a bone graft developed from waste bovine teeth for bone regeneration. Mater. Today Commun..

[19] Timlin J.A., Carden A., Morris M.D., Rajachar R.M., Kohn D.H. (2000). Raman spectroscopic imaging markers for fatigue-related microdamage in bovine bone. Anal. Chem..

[20] Sofronia A.M., Baies R., Anghel E.M., Marinescu C.A., Tanasescu S. (2014). Thermal and structural characterization of synthetic and natural nanocrystalline hydroxyapatite. Mater. Sci. Eng. C.

[21] Londoño-Restrepo S.M., Ramirez-Gutierrez C.F., del Real A., Rubio-Rosas E., Rodriguez-García M.E. (2016). Study of bovine hydroxyapatite obtained by calcination at low heating rates and cooled in furnace air. J. Mater. Sci..

[22] Johnson G.S., Mucalo M.R., Lorier M.A. (2000). The processing and characterization of animal-derived bone to yield materials with biomedical applications. Part 1: Modifiable porous implants from bovine condyle cancellous bone and characterization of bone materials as a function of processing. J. Mater. Sci. Mater. Med..

[23] Rogers K.D., Daniels P. (2002). An X-ray diffraction study of the effects of heat treatment on bone mineral microstructure. Biomaterials.

[24] Lafuente B., Downs R.T., Yang H., Stone N. (2016). The power of databases: The RRUFF project. Highlights in Mineralogical Crystallography.

[25] Teixeira S., Ferraz M.P., Monteiro F.J. (2008). Biocompatibility of highly macroporous ceramic scaffolds: Cell adhesion and morphology studies. J. Mater. Sci. Mater. Med..

[26] Joschek S., Nies B., Krotz R., Göpferich A. (2000). Chemical and physicochemical characterization of porous hydroxyapatite ceramics made of natural bone. Biomaterials.

[27] Karageorgiou V., Kaplan D. (2005). Porosity of 3D biomaterial scaffolds and osteogenesis. Biomaterials.

[28] Richardson C.R., Mellonig J.T., Brunsvold M.A., McDonnell H.T., Cochran D.L. (1999). Clinical evaluation of Bio-Oss^®^: A bovine-derived xenograft for the treatment of periodontal osseous defects in humans. J. Clin. Periodontol..

[29] Sousa S.B., Castro-Silva I.I., Da Rocha Coutinho L.A.C., Lenharo A., Granjeiro J.M. (2013). Osteoconduction and Bioresorption of Bone Allograft versus Anorganic Bovine Bone Xenograft: A Histomorphometric Study in Humans. J. Biomim. Biomater. Tissue Eng..

[30] Renders G.A.P., Mulder L., van Ruijven L.J., van Eijden T.M.G.J. (2007). Porosity of human mandibular condylar bone. J. Anat..

[31] Kuboki Y., Jin Q., Kikuchi M., Mamood J., Takita H. (2002). Geometry of artificial ECM: Sizes of pores controlling phenotype expression in BMP-induced osteogenesis and chondrogenesis. Connect. Tissue Res..

[32] Chu T.M.G., Orton D.G., Hollister S.J., Feinberg S.E., Halloran J.W. (2002). Mechanical and in vivo performance of hydroxyapatite implants with controlled architectures. Biomaterials.

[33] Wang W., Yeung K.W.K. (2017). Bone grafts and biomaterials substitutes for bone defect repair: A review. Bioact. Mater..

[34] Ravi N.D., Balu R., Sampath Kumar T.S. (2012). Strontium-Substituted Calcium Deficient Hydroxyapatite Nanoparticles: Synthesis, Characterization, and Antibacterial Properties. J. Am. Ceram. Soc..

[35] Orlovskii V.P., Komlev V.S., Barinov S.M. (2002). Hydroxyapatite and hydroxyapatite-based ceramics. Inorg. Mater..

[36] Woodard J.R., Hilldore A.J., Lan S.K., Park C.J., Morgan A.W., Eurell J.A.C., Clark S.G., Wheeler M.B., Jamison R.D., Wagoner Johnson A.J. (2007). The mechanical properties and osteoconductivity of hydroxyapatite bone scaffolds with multi-scale porosity. Biomaterials.

[37] Murugan E., Akshata C.R., Ilangovan R., Mohan M. (2022). Evaluation of quaternization effect on chitosan-HAP composite for bone tissue engineering application. Colloids Surf. B Biointerfaces.

[38] Shuai C., Yang W., Feng P., Peng S., Pan H. (2021). Accelerated degradation of HAP/PLLA bone scaffold by PGA blending facilitates bioactivity and osteoconductivity. Bioact. Mater..

